# Health-related quality of life among patients with long COVID according to the presence of a diagnosis of functional somatic disorder: A cross-sectional study

**DOI:** 10.1371/journal.pone.0354238

**Published:** 2026-08-05

**Authors:** Clément Gouraud, Sabrina Guemouni, Patricia Thoreux, Charles Ouazana-Vedrines, Victor Pitron, Corentin Verot, Kewei Xiang, Florian Baudry, Brigitte Ranque, Cédric Lemogne, for the CASPer-COVID Study Group

**Affiliations:** 1 Service de psychiatrie de l’adulte, AP-HP, Hôpital Hôtel Dieu, Paris, France; 2 CIMS (Centre d’Investigations en Médecine du Sport), APHP, Hôpital Hôtel Dieu, Paris, France; 3 Université Paris Cité and Université Sorbonne Paris Nord, Inserm, INRAE, Center for Research in Epidemiology and StatisticS (CRESS), Paris, France; 4 Université Paris Cité, VIFASOM (Vigilance Fatigue Sommeil et Santé Publique), Paris, France; 5 Centre du Sommeil et de la Vigilance-Pathologie professionnelle, APHP, Hôtel-Dieu, Paris, France; 6 Unité CASPer, AP-HP, Hôpital Hôtel-Dieu, Paris, France; 7 Service de Médecine interne, AP-HP, Hôpital européen Georges-Pompidou, Paris, France; Mayo Clinic College of Medicine and Science, UNITED STATES OF AMERICA

## Abstract

**Background:**

Long COVID is associated with poor health-related quality of life (QoL), with substantial interindividual variations. This study aimed to investigate the association between QoL and a diagnosis of functional somatic disorder (FSD) among patients seeking care for long COVID.

**Methods:**

Data were drawn from the CASPer-COVID program, a multidisciplinary tertiary care program for patients with persistent symptoms following COVID-19. QoL was evaluated with the 36-Item Short-Form health survey (SF-36), yielding a Physical Component Summary (PCS) and a Mental Component Summary (MCS). Multivariable linear regression analyses were performed to investigate the associations between PCS or MCS scores and a diagnosis of FSD, adjusting for age, gender, body mass index, comorbidities, hospitalization for acute COVID-19, core persistent symptoms, symptom duration, depressive and anxiety symptoms, and physical activity.

**Results:**

The analyses included 773 patients (median age [interquartile range (IQR)]: 44 [36−55] years; 64% women). QoL was markedly impaired (median PCS [IQR]: 44 [31−60]; median MCS [IQR]: 39 [31−49]). A diagnosis of FSD (76.5% of patients) was not associated with MCS (β [95% CI]: 1.11 [−1.30, 3.53]) or PCS (β [95% CI]: −1.60 [−3.88, 0.67]) scores in adjusted analyses. Lower PCS and MCS scores were associated with higher depressive and anxiety symptoms, lower physical activity levels, and pain. In addition, lower PCS scores were associated with female gender, hospitalization for acute COVID-19 and longer symptom duration.

**Conclusion:**

In patients seeking care for long COVID in a tertiary care setting, QoL did not differ significantly between those diagnosed with FSD and other patients.

## Introduction

Months after an acute infection with SARS-CoV-2, a substantial number of patients continue to experience persistent symptoms, a condition frequently referred to as “Long COVID” [1]. The patient-developed term ‘Long COVID’ corresponds to the World Health Organization’s (WHO) “post-COVID-19 condition”. In its definition, the WHO emphasizes its significant impact on quality of life (QoL) and overall functioning. In this article, we use the term ‘Long COVID,’ because it is more frequently used in clinical practice and by patients themselves. Impaired QoL among patients with Long COVID has been extensively documented, along with its associated factors [2–5]. A systematic review and meta-analysis identified fatigue and history of Intensive Care Unit (ICU) admission as key contributors to poorer QoL [2]. Other studies found several other factors associated with impaired QoL such as the number of persistent symptoms, specific symptoms (including sleep disturbances, cognitive complaints, or anxiety and depressive symptoms), and low levels of physical activity [3–5]. Conversely, positive associations with QoL were found in relation to professional activity, perceived self-efficacy, and patient activation [3,6].

Long COVID is a challenging condition for both clinicians and researchers. Despite extensive investigations into potential pathophysiological pathways, replicated findings remain scarce and no single hypothesis fully explains the heterogeneity of this condition [7]. Long COVID encompasses a diverse array of non-specific symptoms, whose profound impact on patients’ lives often contrasts with the lack of abnormalities identified by physical examination or routine diagnostic tests [8–11]. After careful assessment, many patients with Long COVID meet the criteria for a “functional somatic disorder” (FSD) [12]. This diagnosis is based on both positive arguments (i.e., the presence of cognitive and behavioral mechanisms such as classical conditioning, symptom avoidance, focused attention on bodily functioning, catastrophizing and intolerance to uncertainty) [8,12,13] and negative arguments (i.e., the absence of clinical findings or test results that fully explain the symptoms) [9,13]. This clinically useful categorization differs from the former DSM-IV classification of “somatoform disorders,” which primarily relied on negative diagnostic criteria (i.e., the absence of clinical or paraclinical explanations for the symptoms). It also differs from more recent classifications, such as the DSM-5 somatic symptom disorder or the ICD-11 bodily distress disorder, which focus exclusively on positive criteria (i.e., the presence of excessive thoughts, emotions, and behaviors related to the symptoms). The diagnosis of functional somatic disorders (FSD) involves an approach that combines both positive and negative criteria at the time of assessment. Furthermore, the contribution of cognitive and behavioral mechanisms to symptom persistence does not preclude the involvement of other mechanisms, but widens the scope of therapeutic perspectives within a biopsychosocial approach [8,12,14,15], including cognitive and behavioral therapy, which has shown promise in helping patients with long COVID [16–19].

Unfortunately, although it might be well accepted when carefully explained through individual consultation [13], the diagnosis of FSD is frequently perceived as stigmatizing [7] and may be misinterpreted as a dismissal of the significant QoL impairment experienced by patients [20,21]. Evidence suggests that physicians and patients often diverge in their perceptions of Long COVID’s severity, potentially due to the lack of identifiable physiological abnormalities. Nevertheless, research consistently demonstrates that FSD are associated with substantial QoL impairments, comparable to – or even greater than – those observed in other medical conditions [22–25]. Therefore, the main objective of this study was to investigate factors associated with QoL among patients seeking care for long COVID, with a particular focus on the relationship between QoL and a diagnosis of FSD.

## Methods

### Population

CASPer-COVID (Circuit Ambulatoire de prise en charge des Symptômes Persistants après un épisode de COVID-19) is a multidisciplinary day-hospital evaluation program dedicated to patients with Long Covid [13] in a university hospital in Paris, France. Patients are referred by their general practitioner and are first asked to complete a questionnaire on their medical situation. The medical coordinators of the program (KX and BR) then verify that patients have undergone the symptom-driven medical workup recommended by the Haute Autorité de Santé (HAS) [26], and request additional investigations when necessary (Table 1). Then patients are offered a multidisciplinary day-hospital evaluation consisting of three consecutive one-hour consultations with an internist or infectious disease specialist, a psychiatrist, and an adapted physical activity specialist. Consecutive adult patients (aged >18 years) who attended the program between October 5, 2021 and October 23, 2024 were included. Referrals were primarily regional, with most patients coming from the Paris area. During that period, an evaluation of health-related QoL was systematically performed on the day of the evaluation, with the completion of the 36-Item Short-Form Health survey questionnaire (SF-36). Details about the CASPer-COVID program are available elsewhere [13].

**Table 1:** Medical work-up required before evaluation in the CASPer unit.

**Medical consultations required prior to the day-hospital program**	
	At least one consultation with the general practitioner. At least one consultation with an organ specialist if the predominant symptom refers to a particular organ/system (e.g., cardiologist, pulmonologist, neurologist, rheumatologist, allergist)
**Systematic set of laboratory tests**	
	Complete blood count
	C-reactive protein
	Serum electrolytes, creatinine, and estimated glomerular filtration rate (eGFR)
	Serum calcium, magnesium, and phosphate
	Serum ferritin
	Liver function tests
	Thyroid-stimulating hormone (TSH)
	HIV serology
	
**Complementary tests depending on the symptoms**	
	If there is extreme fatigue: morning serum cortisol, short Synacthen test, and serum protein electrophoresis
	If there is chronic diarrhea: serum tryptase, fecal calprotectin, and coeliac serology
	If there are arthralgias or myalgias: creatine kinase, antinuclear antibodies, anti-dsDNA antibodies, ENA antibodies, and rheumatoid factor
	If there is chest pain: ECG, troponin, and D-dimers; perform CT pulmonary angiography directly in case of O_2_ desaturation
	If there are allergic manifestations: serum tryptase, histamine, total Ig E, serum protein electrophoresis, and complement C3/C4
**Imaging**	
	If there is chest pain or dyspnea: cardiac ultrasound
	If there is persisting chest pain without explanation: cardiac MRI
	If there is chest pain or dyspnea: CT pulmonary angiography
	If there are central neurological features, including cognitive impairment: brain MRI
**Functional tests**	
	If there is dyspnea: respiratory function tests, arterial blood gas analysis, and 6-minute walk test
	If there is paresthesia or peripheral motor or sensory deficit: electromyography
	If obstructive sleep apnea is suspected: polysomnography
	If there is gastroesophageal reflux disease, epigastric pain, nausea or vomiting: upper gastrointestinal endoscopy
	If there is chronic diarrhea: colonoscopy

### Main outcome

The SF-36 yields a physical component summary (PCS) and a mental component summary (MCS) with higher scores (from 0 to 100) indicating higher levels of quality of life.

The Minimum Clinically Important Difference (MCID) for the SF-36 is frequently defined as a difference >2.5 points for each component (or >5 points for the total score) [27–29].

### Explanatory variables

We considered several variables extracted from the medical records as potential confounders of the association between FSD diagnosis and QoL: age, gender, variables depicting medical history (history of cardiovascular or pulmonary disease, history of anxiety or depressive disorder), body mass index, hospitalization for COVID-19 (as a proxy of the episode severity), duration of the persistent symptoms and presence of the core Long Covid symptoms (i.e., fatigue, breathlessness, cognitive dysfunction, pain), level of physical activity (Ricci & Gagnon scale: nine items scoring 1–5, a score <18 indicating low physical activity), depressive and anxiety symptoms (Hospital Anxiety and Depression Scale (HADS), 14 items scoring 0–3, yielding two subscores with a score ≥ 8 indicating high levels of anxiety or depression). We also recorded whether or not a diagnosis of FSD was established. To establish the diagnosis of FSD, all patients first underwent a standardized, symptom-driven medical workup (see Table 1) and a clinical evaluation by an internist or an infectious diseases specialist. FSD was considered only when this workup did not identify clinical findings or test results sufficient to fully explain the persistent symptoms. Patients then underwent a one-hour psychiatric consultation with a psychiatrist trained in FSD. The diagnosis of FSD was based on the identification of cognitive and behavioral mechanisms consistent with FSD, together with the absence of medical findings fully accounting for the symptoms. The identification of cognitive and behavioral mechanisms followed a structured symptom assessment grid (see S1 Table). Ongoing data completion from medical records is routinely performed in the CASPer-COVID Program. For these analyses, data from medical records were processed from January 2023 (01/01/2023) and analyzed in March 2026 (from 01/03/2026 to 29/03/2026) and all data have been anonymized before analyses.

### Statistical analysis

First, we conducted descriptive analysis using percentages for categorical variables and median and interquartile ranges for continuous variables.

Then, we computed multivariable linear regression models with the SF-36 PCS or MCS scores as the outcome. To address potential confounding, we used two sequential models. Model 1 included pre-COVID characteristics (age, gender, body mass index, history of cardiovascular or pulmonary disease, and history of depressive or anxiety disorder). Model 2 was further adjusted for hospitalization for acute COVID-19, duration of persistent symptoms, core long COVID symptoms, depressive and anxiety symptoms, physical activity level, and FSD diagnosis. Multivariable analyses were performed on complete cases, i.e., participants with no missing data for the outcome and covariates included in each model. To ease clinical interpretation, HADS subscales (HADS-A and HADS-D) were divided by the interquartile interval (i.e., the difference between the 25th and the 75th percentile) of each scale in this population.

Because the multivariable linear regression analyses were based on complete cases, we compared the descriptive characteristics of complete-case participants and those with missing data to limit potential selection bias.

As a sensitivity analysis, we explored whether replacing the FSD diagnosis with the SSD-12 changed the observed association. The SSD-12 is a scale designed to assess the cognitive features of the DSM-5 somatic symptom disorder B criteria.

All data analyses were performed using Stata 15.0 (StataCorp, College Station, TX). The significance threshold was set at two-tailed *p* < 0.05.

### Ethics statement

This retrospective study was designed in accordance with the declaration of Helsinki. The project received authorization from the “Comité d’éthique de la recherche AP-HP Centre” (CERAPHP); IRB registration #00011928, Ref 2022-10-08 [13]. Written consent was not required, considering that this study consists in a retrospective chart review observational study, in accordance with French law n◦2012–300 of March 5, 2012 regarding research involving human subjects. Nevertheless, participants are informed that medical records can be used for research purposes via the medical examination report they receive after their participation. Participants were also informed that they could object to the use of their data.

## Results

A total of 773 participants were included in the analyses (Table 2). As expected, QoL scores were fairly low with a median PCS score of 44 (IqR 31–60) and a median MCS score of 39 (IqR 31–49).

**Table 2:** Characteristics of the population (N = 773).

CONTINUOUS VARIABLES	median	IqR
**Age**	45	36–55
**SF-36 PCS** (n = 725)	44	31–60
**SF-36 MCS** (n = 724)	39	31–49
**Duration of persistent symptoms** (in weeks, n = 749)	84.3	44–123.3
**HADS-A** (n = 740)	9	6–13
**HADS-D** (n = 740)	8	5–12
**Ricci & Gagnon Score** (n = 751)	14	10–24
**CATEGORICAL VARIABLES**	**n**	**%**
**Gender (women)**	496	64.2
**Hospitalization for acute COVID-19** (n = 770)	90	11.7
**History of cardiovascular or pulmonary disease** (n = 770)	165	21.4
**History of depressive or anxiety disorder** (n = 768)	251	32.7
**Body mass index** (n = 743)		
< 18.5	53	7.1
≥ 18.5 & < 25	395	53.2
≥ 25 & < 30	192	25.8
≥ 30	103	13.9
**Core long COVID persistent symptoms**		
Fatigue (n = 770)	644	83.6
Breathlessness (n = 765)	321	42.0
Cognitive complaints≥ (n = 767)	379	49.4
Pain (n = 768)	434	56.5
**Diagnosis of functional somatic disorder** (n = 759)	581	76.5

HADS-A: Hospital Anxiety and Depression – Anxiety subscale; HADS-D: Hospital Anxiety and Depression – Depression subscale; IqR: Interquartile Range; MCS: mental component summary; PCS: physical component summary; SF-36: Short-Form Health survey questionnaire; Missing data due to incomplete medical records.

Table 3 presents the results of the multivariable linear regression models. As a result of missing data in some covariates, the sample size differed across models. In addition to the expected negative association with depressive and anxiety symptoms, the MCS score was also associated negatively with pain and positively with the level of physical activity. Likewise, the PCS score was also negatively associated with depressive and anxiety symptoms and pain, and positively with physical activity level. In addition, female gender, hospitalization for acute COVID-19 and duration of persistent symptoms were negatively associated with PCS.

**Table 3:** Variables associated with SF-36 score in multivariable linear regression models.

	SF-36 Mental Component Summary score						SF-36 Physical Component Summary score					
	Model 1 (N = 694)			Model 2 (N = 643)			Model 1 (N = 693)			Model 2 (N = 642)		
	β	95% CI	p	β	95% CI	p	β	95% CI	p	β	95% CI	p
**Age**	0.01	−0.10; 0.12	0.85	0.01	−0.07; 0.09	0.83	0.02	−0.07; 0.11	0.67	0.03	−0.05; 0.11	0.47
**Gender (women)**	1.49	−1.35; 4.33	0.30	0.77	−1.42; 2.95	0.49	**−3.74**	**−6.04; −1.45**	**<0.01**	**−3.18**	**−5.23; −1.12**	**<0.01**
**BMI < 18.5**	−0.40	−5.78; 4.97	0.88	−0.32	−4.32; 3.67	0.87	−3.93	−8.27; 0.40	0.08	−3.15	−6.91; 0.61	0.10
**BMI 18.5–24.9**	Ref.			Ref.			Ref.			Ref.		
**BMI 25–29.9**	−0.10	−3.31; 3.11	0.95	2.09	−0.39; 4.57	0.10	−2.05	−4.64; 0.54	0.12	0.25	−2.08; 2.58	0.84
**BMI** ≥ **30**	**−6.77**	**−10.9; −2.64**	**<0.01**	−1.03	−4.30; 2.23	0.53	**−6.32**	**−9.66; −2.99**	**<0.01**	−1.04	−4.11; 2.03	0.51
**History of cardiovascular or pulmonary disease**	1.43	−1.92; 4.78	0.40	1.02	−1.52; 3.55	0.43	−1.11	−3.82; 1.61	0.42	−1.20	−3.60; 1.20	0.33
**History of depressive or anxiety disorder**	**−5.76**	**−8.61; −2.90**	**<0.01**	−1.80	−3.98; 0.39	0.11	−2.09	−4.40; 0.21	0.08	0.06	−2.00; 2.11	0.96
**Hospitalization for acute COVID-19**	–	–	–	−2.18	−5.56; 1.21	0.21	–	–	–	**−4.24**	**−7.43; −1.06**	**<0.01**
**Fatigue**	–	–	–	−1.14	−3.95; 1.67	0.43	–	–	–	−0.40	−3.05; 2.24	0.76
**Breathlessness**	–	–	–	−0.84	−2.95; 1.26	0.43	–	–	–	−1.93	−3.92; 0.05	0.06
**Cognitive complaints**	–	–	–	−0.88	−2.94; 1.18	0.40	–	–	–	−0.91	−2.84; 1.03	0.36
**Pain**	–	–	–	**−3.49**	**−5.56; −1.42**	**<0.01**	–	–	–	**−5.47**	**−7.42; −3.52**	**<0.01**
**Duration of persistent symptoms (per week)**	–	–	–	−0.01	−0.03; 0.01	0.24	–	–	–	**−0.02**	**−0.04; 0.00**	**0.04**
**HAD-A**	–	–	–	**−8.12**	**−10.01; −6.22**	**<0.01**	–	–	–	**−3.52**	**−5.30; −1.73**	**<0.01**
**HAD-D**	–	–	–	**−12.06**	**−14.03; −10.09**	**<0.01**	–	–	–	**−6.19**	**−8.04; −4.33**	**<0.01**
**Ricci & Gagnon Score**	–	–	–	**0.16**	**0.04; 0.29**	**0.01**	–	–	–	**0.35**	**0.23; 0.47**	**<0.01**
**Functional somatic disorder**	–	–	–	1.11	−1.30; 3.53	0.37	–	–	–	−1.60	−3.88; 0.67	0.17

BMI: Body Mass Index; CI: Confidence Interval; HAD-A: Hospital Anxiety and Depression – Anxiety subscale; HAD-D: Hospital Anxiety and Depression – Depression subscale.

A diagnosis of FSD was not significantly associated with either MCS (β [95% CI]: 1.11 [−1.30, 3.53]) or PCS score (β [95% CI]: −1.60 [−3.88, 0.67]).

In sensitivity analyses, replacing the FSD diagnosis with the SSD-12 score showed a significant association with both mental and physical quality of life (β = −0.42, 95% CI [−0.56 to −0.29], p < 0.01; and β = −0.56, 95% CI [−0.69 to −0.44], p < 0.01, respectively). The other observed associations did not materially change.

No significant differences were observed in descriptive characteristics between the complete-case population and the population of participants with at least one missing data, except for a higher median HADS-D score among patients with missing data (m = 10 (IqR:6–14) and m = 8 (IqR:5–12); Kruskal wallis test H = 6.49; p = 0.01)

## Discussion

In patients with long COVID attending a tertiary care structure, we found no statistically significant association between a diagnosis of FSD and health-related quality of life at the time of assessment. These findings do not support the assumption that patients diagnosed with FSD have better quality of life than other patients with long COVID. In our regression analyses, the coefficients observed for the association between FSD diagnosis and SF-36 scores were small in magnitude and below the MCID. However, the confidence interval for PCS includes both trivial effects and potentially clinically meaningful effects. Accordingly, the absence of a statistically significant association should not be interpreted as evidence of equivalence, and this observational study cannot distinguish between true equivalence and insufficient precision to exclude a moderate effect.

Interestingly, depressive and anxiety symptoms, and physical activity levels were associated with both mental and physical component scores. Depressive and anxiety symptoms showed particularly large effect sizes, especially for MCS, with decreases of 8 and 12 points across the interquartile ranges of HADS-A and HADS-D, respectively, corresponding to decreases of 0.50 and 0.88 points in the SF-36 score for each 1-point increase in HADS-A and HADS-D, respectively. They were also strongly associated with PCS, with decreases of 3.5 and 6.0 points across the interquartile ranges of HADS-A and HADS-D, respectively, corresponding to decreases of 1.16 and 1.72 points in the SF-36 score for each 1-point increase in HADS-A and HADS-D, respectively. This result is consistent with the conclusion of other studies [2,3,5], although we cannot conclude on the direction of this association. Pain was also associated with worst level of QoL among the physical component score, with poorer PCL, with a difference exceeding the MCID, whereas the other core symptoms were not. The absence of an association between fatigue or cognitive complaints and MCS may appear surprising. However, in this tertiary-care cohort of severely affected patients, these symptoms were highly prevalent and may therefore have had limited discriminative value. In addition, the mental component of the SF-36 may be more strongly influenced by broader emotional distress than by the specific burden of fatigue or cognitive complaints, which may explain the stronger associations observed with depressive and anxiety symptoms. Longer symptom Duration of persistent symptoms and hospitalization for acute episode COVID-19 were also associated poorer PCS; for hospitalization, the magnitude of the association exceeded with a clinically relevant difference for hospitalization, above the MCID, which is consistent with other previous studies [2]. Finally, we found that female gender was associated with worst level of the physical component score. Interestingly, a history of depressive or anxiety disorder was associated with lower MCS score in model 1 but not after full adjustment. This attenuation may reflect an overlap with current depressive and anxiety symptoms included in model 2.

The main result of our study is that health-related QoL of patients did not differ significantly between patients with long COVID who were diagnosed with FSD by an experienced multidisciplinary team and other patients with long COVID.

Indeed, it has already been described in other fields that FSD were associated with altered quality of life [22,23,30], in a comparable manner to well-defined medical conditions with similar symptoms [24,30], and in some instances, even more so [22,23,25]. It has also been extensively described that cognitive mechanisms observed in FSD as well as in the DSM-5 “Somatic Symptom Disorder” were associated with poor QoL [31], even when another pathology has been clearly identified [32]. Furthermore, the research field and development of therapeutic strategies for FSD places special emphasis on QoL [33].

In sensitivity analyses, replacing FSD diagnosis with the SSD-12 score found a significant association between the SSD-12 score and QoL. However, this result should be interpreted with caution. Because the SSD-12 captures symptom-related perceived impairment, its association with health-related QoL may reflect substantial conceptual overlap between predictor and outcome. This may increase its apparent predictive value for a self-reported outcome such as health-related QoL, without necessarily indicating that it is a superior construct to the clinical diagnosis of FSD. In addition, the SSD-12 captures only part of the construct underlying the clinicals diagnosis of FSD, particularly symptom-related thoughts such as negative expectations or perceived severity, whereas it less fully captures behavioral features, such as avoidance and other processes such as heightened vigilance toward bodily sensations. Our data therefore support the idea that the SSD-12 may provide complementary information, but they do not allow conclusions about incremental predictive validity beyond the clinical diagnosis.

Our study as some strength as it relies on a clinical population with severe long COVID symptoms, a multidisciplinary evaluation that allowed a comprehensive evaluation of medical condition and mental health, including a systematic search for positive arguments in favor of a diagnosis of FSD. Nevertheless, this study also has limitations. First, the cross-sectional nature of the study prevents causal interpretation of the results. For example, decreased physical activity could reflect avoidant behavior towards physical activity (as a frequent characteristic of FSD), and could therefore contribute to poor QoL, or could be a consequence of physical impairment. Depressive symptoms could also, at least in part, reflect the intensity of psychological distress associated with FSD, thereby capturing a similar dimension. Furthermore, because our sample was drawn from a tertiary care center, referral bias cannot be excluded, which may limit the generalizability of our findings. Indeed, the long median duration of persistent symptoms (about 1.6 years) likely reflects the inclusion of particularly chronic and refractory cases. Although we do not have comparative data to determine whether this duration is typical of tertiary care long COVID programs, it suggests that our findings may not apply to patients with milder long COVID managed in primary care or to those with less severe functional impairment who would not require evaluation in a specialized center. Nevertheless, given that health-related QoL was our outcome, studying a cohort of severely affected patients remains clinically meaningful. In addition, only a small proportion of our sample had severe acute COVID-19, and factors associated with QoL may differ in patients with residual organ damage following more severe disease. Finally, interrater agreement for the FSD diagnosis was not formally assessed, and the data collected did not allow calculation of kappa coefficients. However, the participating psychiatrists all belonged to the same department and received the same initial theoretical and practical training, including standardized training sessions before starting the assessments. In addition, frequent clinical meetings were held to discuss clinical subtleties and diagnostic difficulties, with the aim of improving diagnostic harmonization.

To conclude, in patients with long COVID seeking care in a tertiary care setting, a diagnosis of FSD by an experienced multidisciplinary team was not significantly associated with health-related QoL. Therefore, the diagnosis of FSD in this context should be acknowledged as the recognition of a severe condition. This could reduce the stigma that patients with this condition often experience [34,35], and thus reduce barriers to receive effective care targeting mechanisms that long COVID may share with FSD [16–19].

## Supporting information

S1 Table Clinical arguments supporting the diagnosis of functional somatic disorder. (DOCX)
