# Supplementary material for: Health-related quality of life among patients with long COVID according to the presence of a diagnosis of functional somatic disorder: A cross-sectional study
Source: PLoS One. 2026 Aug 5;21(8):e0354238. doi: 10.1371/journal.pone.0354238 (PMC13440821; doi:10.1371/journal.pone.0354238)
Supplement: Supplementary file 1 [file pone.0354238.s001.docx]

**Supplementary table 1: Clinical arguments supporting the diagnosis of functional somatic disorder**

| **Core clinical features** |  |
| --- | --- |
|  | Persistent and troublesome physical symptoms associated with functional impairment |
|  |  |
| **Negative arguments** |  |
|  | Absence of clinical findings or test results that fully explain the symptoms, based on the algorithm presented in table 1 and the comprehensive clinical evaluation performed by the internist or infectious disease specialist during the day-hospital assessment |
|  |  |
| **Positive arguments suggestive of cognitive and behavioral mechanisms** |  |
|  | Features suggestive of conditioned symptom reactivation  - Similarity between the initial and the persistent symptoms  - Repeated triggering or exacerbation of persistent symptoms in contexts similar to those in which they first appeared or worsened |
|  | Behavioral manifestations of symptom avoidance  - Avoidance driven by the burden of the symptoms per se  - Avoidance driven by fear of worsening the symptoms  - Avoidance driven by fear of worsening the condition |
|  | Excessive attention to bodily functioning  - Hypervigilance to bodily sensations, including sensations beyond the persistent symptoms themselves  - Repeated reports of symptom relief when attention is directed away from bodily functioning |
|  | Catastrophizing  - Attribution of symptoms to a serious but as yet undetected medical condition  - Negative expectations regarding the expected duration and course of the symptoms |
|  | Intolerance of uncertainty  - Lack of reassurance despite normal clinical findings and test results  - Repeated health-related internet searches and specialist consultations despite normal clinical findings and test results |
